# Paclitaxel/oxaliplatin/fluorouracil (TOF) regimen versus S-1/oxaliplatin (SOX) regimen for metastatic gastric cancer patients

**DOI:** 10.18632/oncotarget.13721

**Published:** 2016-11-30

**Authors:** Xichao Dai, Xizhi Zhang, Chaomin Wang, Jingting Jiang, Changping Wu

**Affiliations:** ^1^ Department of Oncology, Subei Peoples Hospital of Jiangsu Province, Clinical Medical College of Yangzhou University, Jiangsu, Yangzhou, China; ^2^ Department of Tumor Biological Treatment, The Third Affiliated Hospital of Soochow University, Suzhou, China; ^3^ Department of Oncology, The Third Affiliated Hospital of Soochow University, Suzhou, China

**Keywords:** paclitaxel, oxaliplatin, fluorouracil, S-1, metastatic gastric cancer

## Abstract

**Aims and background:**

This study was designed to compare the efficacy and safety of paclitaxel/oxaliplatin/fluorouracil (TOF) regimen and S-1/oxaliplatin (SOX) regimen for metastatic gastric cancer (GC) patients.

**Methods:**

Sixty patients were divided into TOF group and SOX groups randomly. Patients in the TOF group received paclitaxel (135 mg/m^2^ iv) on day 1, oxaliplatin (100 mg/m^2^ iv) on day 1, fluorouracil (500 mg/m^2^ continuous iv) on day 1-5. The patients in the SOX group received oxaliplatin (130 mg/m^2^ iv) on day 1 and S-1 (40 mg~60mg orally twice/day based on body surface area) on days 1-14. All the treatments were repeated every 21d for 4-6 cycles.

**Results:**

The ORR and DCR of TOF group was 43.3% and 60.0%, respectively while that of SOX group was 36.7% and 56.7%. There were no statistical differences between the ORRs (?^2^ = 0.278) and the DCRs (?^2^ = 0.069) of the 2 groups. The majority of adverse events of two groups were hematological and digestive ones. Most of them were grade I and II. The adverse event rate of TOF group was higher than SOX group. The PFS times of TOF and SOX groups were 6.5 and 5.8 months, respectively. There was no statistical difference between the PFSs of the 2 groups (*P* = 0.451).

**Conclusions:**

The efficacies of TOF and SOX regimens are similar but the safety of SOX regimen better than TOF regimen.

## INTRODUCTION

Gastric cancer occupies the second place of deaths caused by malignances over the world and It is especially popular in Asia [1–3],which is the third common carcinoma in China [4]. and the incidence rate and death rate of gastric cancer in Jiangsu Province are especially higher than the national average [5].

Surgical resection is the preferred treatment for gastric cancer, but approximately two-thirds of patients have metastatic disease at the time of diagnosis [6]. Prognosis in these patients is adverse. Median survival time of them is 3 to 5 months without treatment [7] while 5-year survival rate is reported as 9.4% [8]. Local recurrence and distant metastasis occur in 60% of mGC patients even with a radical stomach surgery [9]

Chemotherapy is the most effective treatment option for patients with advanced gastric cancer who can’t be operated on [10–12] and the efficacy of postoperative chemotherapy has been acknowledged [13]. However, a worldwide consensus on standard chemotherapy regimens has yet to be established. The prognosis has gradually improved because of advances in chemotherapy regimens, but is not yet satisfactory.

Among various regimens, the combinations of paclitaxel/oxaliplatin/fluorouracil (TOF) regimen and S-1/oxaliplatin (SOX) regimen have become two important ones.

. As we all knew,the combination of Docetaxel /cisplation/ fluorouracil (DCF) had been verified by V325 trail to have higher RR, TTP, OS than FP4W [14].

Oxaliplatin is the third generation of diaminocyclohexane platinum compounds with broad spectrum antitumor activity, which is better safety than cisplatin [13, 15]. Paclitaxel is another Taxanes besides Docetaxel which can induce hyperstabilization of microtubules resulting in cell cycle arrest and apoptosis [16, 17]. The objective response rate of patients with gastric cancer to paclitaxel is 20%-25% [18]. So We assumpt that the combination of paclitaxel/oxaliplatin/fluorouracil (TOF) regimen may substitute for DOF with lower toxicity.

S-1 is an oral anti-cancer drug of modified Fluorouracil composed of oteracil potassium, 5-chloro-2,4-dihydroxypy-ridine, tegafur [19],. which was also demonstrated to be not inferior to intravenous infusion of 5-FU when both were administered as a single drug [20, 21]. Combination of cisplatin with S-1(CS) is considered as standard first-line chemotherapy regimen for metastatic gastric cancer by SPIRIT trail in Japan [21, 22].

The applying of S-1 as adjuvant chemotherapy for mGC can improve the overall survival (OS) and relapse-free survival [23].

OS in mGC was proven to benefit from S-1 based chemotherapy rather than 5-FU-based chemotherapy in a metal-analysis [3, 24]. Combination of S-1 with oxaliplatin(SOX) was demonstrated not to inferior to S1 plus cisplatin(CS) in PFS. SOX regimen also had well tolerance in patients with mGC [25]. From this, SOX is thought as a new first-line treatment choice of mGC in Asia, especially in China and Japan [21].

But, What is the activity of combinations of paclitaxel/oxaliplatin/fluorouracil (TOF) regimen in mGC ?

It is well known that only one regimen is optimal at one time. UP to now,There is no study comparing efficacy and safety of SOX. and TOF. So, This randomized and controlled study was performed to compare efficacy and safety of two regimen in mGC patients.

## PATIENTS AND METHODS

### Patients

This study was approved by the ethics committees of all participating medical institutions and conducted according to the principles of the Declaration of Helsinki and Good Clinical Practice Guidelines. Between Feb. 2012 and Jan. 2014, a total of 60 patients bearing mGC were enrolled. All patients gave their written informed consent before enrollment.

The inclusion criteria were 1) pathologically confirmed mGC (stage IV), 2) ages between 20 and 80 years, 3) measurable or assessable lesions by imaging studies according to the RECIST guideline [26], 4) no prior chemotherapy except for postoperative adjuvant chemotherapy for more than 12 months before entry into the study, 5) Eastern Cooperative Oncology Group (ECOG) performance status score less than 3, 6) hepatic function (total bilirubin ≤ 1.5 × the institutional upper limit of normal value, aspartate aminotransferase/alanine aminotransferase ≤ 2.5 × the institutional upper limit of normal value, and alkaline phosphatase ≤ 2.5 × the institutional upper limit of normal value), renal function (serum creatinine level ≤ 1.5 mg/dL and creatinine clearance ≥ 50 ml/min) and adequate bone marrow function (hemoglobin level ≥ 90 g/L, white blood cell count of 4-10×10^9^/L, neutrophil count ≥ 2×10^9^/L, and platelet count ≥ 100×10^9^/L), and 7) estimated life expectancy more than 3 months and 8) no other secondary malignant tumors.

The exclusion criteria were 1) preexisted peripheral toxicity ≥ grade 2 of the National Cancer Institute Common Toxicity Criteria, 2) concurrent or prior malignancy, 3) central nervous system metastases, 4) concurrent treatment that interfered with the study evaluation, 5) active infection, 6) other uncontrolled underlying medical conditions that would impair the ability of the patients to receive the planned treatment, 7) having inadequate calorie and fluid intake, and 8) pregnant, and breastfeeding women or women of child-bearing potential without adequate contraception.

### Treatment

The patients were divided into TOF group and SOX groups randomly. Patients in the TOF group received paclitaxel (135 mg/m^2^ iv) on day 1, oxaliplatin (100 mg/m^2^ iv) on day 1, fluorouracil (500 mg/m^2^ continuous iv) on day 1-5. The patients in the SOX group received oxaliplatin (130 mg/m^2^ iv) on day 1 and S-1 (40 mg twice/day for body surface area < 1.25 m^2^ and 60 mg twice/day for body surface area between 1.25 and 1.50 m^2^ orally) on days 1-14. All the treatments were repeated every 21 d for 4-6 cycles.

The dose was modified based on the hematologic parameters and the degree of non-hematologic toxicities. The dose was modified for the TOF group as following, 1) if the hepatotoxicity reached grade 2 or more, the dose of paclitaxel for the following treatment was reduced to 100 mg/m^2^ on days 1; if the hepatotoxicity was grade 3/4, the study was discontinued. (2) If the bone marrow suppression reached grade 4, the dose of paclitaxel for the following cycle was reduced to 100 mg/m^2^ on day 1; if the bone marrow suppression reached grade 4, the study was discontinued; 3) if the mucositis reached grade 3/4, fluorouracil was administered from the next cycle for only 3 days; 4) if the creatinine clearance rate decreased to 30-50 mL/min resulted by the nephrotoxicity, the dose of oxaliplatin was reduced by 50%; if the creatinine clearance rate was lower than 30 mL/min, the study was discontinued.

The dose was modified for the SOX group as following: 1) if the neurotoxic toxicity was grade 1/2, the dose of oxaliplatin was reduced by 25%; 2) if the neurotoxic toxicity was grade 3/4 or persistent, the oxaliplatin was omitted from the regimen until the neurotoxic toxicity was resolved to grade 1 or better.

### Evaluation

The outcomes of electrocardiogram, computed tomography (CT) scan, and levels of tumor markers (CA19-9, CA72-4, CA24-2 and carcinoembryonic antigen) were obtained from the patients within 7 d after enrollment. Hematology tests, biochemistry tests, and assessment of symptoms and signs were carried out for the patients within 3 days before enrollment and every week during the study period. CT scans were carried out and levels of tumor markers were measured before each cycle. According to the RECIST guideline [16], responses concluded complete response (CR), partial response (PR), stable disease (SD), and progressive disease (PD). To confirm the PR or CR, the levels of tumor markers were measured no less than 4 weeks after the objective response was obtained. Responses were assessed by the independent review committee. The overall response rate (ORR) was defined as the sum of CR and PR rates. The disease controlled rate (DCR) was defined as the sum of CR, PR and SD rates. Safety was evaluated according to the NCI-CTC. PFS is defined as the time from randomization until objective tumor progression or death.

PFS is primary endpoint of our study. The secondary endpoint is ORR, DCR.

### Statistical analysis

Statistical analysis was performed with the SPSS software (version 17.0, SPSS). Chi-square test was used to compare the categorical data. *P* < 0.05 was considered statistically significant.

## RESULTS

### Patient baseline characteristics

The patient baseline characteristics are listed in Table 1. The difference between two groups was not statistically significant in any characteristics.

**Table 1:** Patients baseline characteristics (*n* = 60)

Characteristics	Patients (%)		*P*
	TOF (*n*= 30)	SOX (*n*= 30)	
Sex			0.785
Female	9	11	
Male	21	19	
Age (years)			0.787
Median	58	57	
Range	21-73	20-75	
Histologic type			0.757
Adenocarcinoma	22	21	
Adenosquamous carcinoma	5	7	
Mucinous carcinoma	3	2	
No. of metastatic lesion			0.771
0-1	7	9	
≥ 2	23	21	
Prior adjuvant chemotherapy			1.000
No	18	18	
Yes	12	12	

### ORR and DCR

The patients all received 4-6 cycles of chemotherapy and were suitable for response evaluation.

No patients developed severe adverse events leading to exclusion of the efficacy analysis.

Among the patients in TOF group, 1 achieved a CR, 12 achieved a PR, 5 achieved a SD, and 12 achieved a PD, with an ORR of 43.3% and a DCR of 60.0%.

Among the patients in SOX group, 1 achieved a CR, 10 achieved a PR, 6 achieved a SD, and 13 achieved a PD, with an ORR of 36.7% and a DCR of 56.7%. There were no statistical differences between the ORRs (χ^2^ = 0.278) and the DCRs (χ^2^ = 0.069) of the 2 groups. See Table 2.

**Table 2:** Objective response of TOF and SOX groups, *n* (%)

Group	CR	PR	SD	PD	ORR		DCR	
TOF (*n* =30)	1 (3.3)	12 (40.0)	5 (16.7)	12 (40.0)	24 (80.0)	*P* = 0.243 χ2 = 1.367	18 (60.0)	*P* = 0.793 χ2 = 0.069
SOX (*n* =30)	1 (3.3)	10 (33.3)	6 (20.0)	13 (43.3)	20 (66.7)		17 (56.7)	

In TOF group, the efficacy of the 1 patient was evaluated as CR, 12 patients was evaluated as PR. 5 patients was evaluated as SD, 12 patients was evaluated as PD. The ORR was 43.3% with a DCR of 60.0%. In SOX group, the efficacy of the 1 patient was evaluated as CR, 10 patients was evaluated as PR. 6 patients was evaluated as SD, 13 patients was evaluated as PD. The ORR was 36.7%, with a DCR of 56.7%. There were no statistical differences between the ORRs (χ^2^ = 0.278) and the DCRs (χ^2^ = 0.069) of the 2 groups. See Table 2.

### Safety

No patient discontinued or ended the treatment due to intolerable adverse events. The majority of adverse events of both the 2 groups were hematological and digestive ones. Most of them were grade I and II and grade III and IV hematological adverse events were also observed. Generally, the adverse event rate of TOF group was higher than SOX group. There were no statistical differences between the grade III and IV adverse events the 2 groups. And basically the adverse events relieved after symptomatic treatment. See Table 3.

**Table 3:** Adverse events of TOF and SOX groups, *n* (%)

Toxicity	TOF		SOX	
	I - II	III - IV	I - II	III - IV
WBC decreasing	27	2	12	0
Neutrophil decreasing	26	2	10	0
Anemia	1	0	1	0
PLT decreasing	8	0	5	0
Diarrhea	10	0	2	0
Vomiting	19	2	10	0
Alopecia	28	0	5	0
Oral mucositis	12	0	2	0
Hand-foot syndrome	7	0	5	0
Neurotoxicity	6	0	5	0

WBC: white blood cell; PLT: platelet

### Progression free survival (PFS)

**Figure 1:**
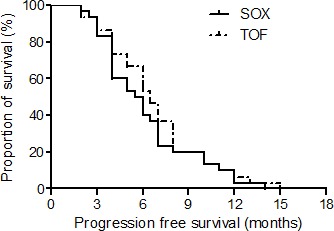
The progression free survival curves of the TOF and SOX groups There is no statistical difference between the progression free survival times of the 2 groups.

The PFS times of TOF and SOX groups were 6.5 and 5.8 months, respectively. There is no statistical difference between the PFSs of the 2 groups (*P* = 0.451). See Figure 1.

## DISCUSSION

Chinese gastric cancer patients are oftern diagnosed at later stage with distant metastases [27] Although there are many treatment modality such as operation combined with chemotherapy which can be used to treat gastric cancer [28, 29], there is currently no standard regimen for mGC patients. Therefore, it is urgent to obtain a new therapeutic strategy with better efficacy and tolerable toxicity. In the present study, SOX regimen is usually considered to be the first-line therapy choice for mGC in China [30] and Japan [31] TOF regimen is not that widely applied. V325 trail has proven the ORR(36.7%), TTP(5.6m) of DCF regimen, docetaxel/cisplatin/ fluorouracil, higher than FP4w [14]. It can be chose as first-line regimen for mGC but for high toxicity.

Following promising phase II study results [32], [33, 34], oxaliplatin was verified to have the non-inferiority compared with cisplatin with low toxicity. As we all know Paclitaxel is another taxane which has been used widely in mGC.

So We assumpted that TOF may substitute for DCF with tolerable side effect.

The efficacy and safety of TOF and SOX regimen were discovered in mGC. We found that the therapeutic efficacies of the 2 groups were similar but SOX regimen seemed to have lower toxicity than TOF regimen.

Different combination chemotherapy regimens as first-line therapy demonstrated median PFS of 5-6 months and response rate of 35-45% [35].

Our data on antitumor response basically meet with this range and we gained a slightly longer PFS.

There are few articles focusing on TOF in mGC, which is possible due to the consideration of the adverse events of this regimen. Actually our results do confirm this consideration.

We did not report the outcome of overall survival of the patients due to the 5-year follow-up has not been finished. And there is no statistical difference between Progression free survival times of the 2 groups. However, we can still find the tendency of longer survival time of TOF regimen. The survival outcome needs to be clarified by studies with larger sample sizes.

Several recent clinical trails have shown that SOX regimen is a effective and easily tolerated treatment method for mGC patients [12, 31, 36, 37].

The response rates of SOX for mGC range from 53.7% to 59.0% [12, 31, 37, 38]. Besides, Koizumi et al. [31] reported the median PFS time of patients receiving SOX regimen was 6.5 months and the incidence of grade 3/4 toxicity was lower. Similarly, Oh et al. [37] and Liu et al. [30] also showed that the SOX regimen was easily tolerated and more convenient treatment as a first-line therapy for mGC patients. All these results met ours.

Moreover, the development of adverse events had worse outcomes than the patients who did not experience adverse events, suggesting that the frequency of adverse events is an important factor in evaluating the efficacy of the drug [39].

Conclusively, we demonstrated that the therapeutic efficacies of TOF regimen and SOX regimen are similar but the safety of SOX regimen is more tolerable than TOF regimen. Hence, we would rather recommend SOX regimen for mGC patients not only because of the acceptable benefits to the efficacy and PFS but more importantly the better safety. Further studies are guaranteed to corroborate the result of this study.
